# Genetic Vulnerability to Experiencing Child Maltreatment

**DOI:** 10.3389/fgene.2019.00852

**Published:** 2019-09-17

**Authors:** Patrizia Pezzoli, Jan Antfolk, Alexander S. Hatoum, Pekka Santtila

**Affiliations:** ^1^Department of Psychology, Åbo Akademi University, Turku, Finland; ^2^Department of Psychiatry, Washington University in St. Louis, St. Louis, MO, United States; ^3^New York University Shanghai, Shanghai Shi, China

**Keywords:** child victimization, gene-environment correlation, sex differences, child sexual abuse, child maltreatment, adverse childhood experiences, heritability, genetics

## Abstract

Although biological factors may influence the risk of experiencing negative life events, the role of genes in the vulnerability to child victimization remains poorly understood. In a large population-based Finnish sample (N = 13,024), we retrospectively measured multiple experiences of child victimization and, in a subsample of twins (n = 9,562), we estimated the extent to which genetic and environmental factors influenced these experiences. In particular, we investigated whether genetic and environmental influences varied depending on the type of child victimization, the genetic relatedness with the perpetrator, and the sex of the victim. Our quantitative genetic analyses supported the presence of both genetic and environmental influences on the occurrence and co-occurrence of child abuse and neglect. We also identified one common etiological pathway underlying multiple child victimizations, and after accounting for this common etiology, we singled out risk factors specific to sexual abuse. Environmental factors shared and nonshared between twins raised together influenced the risk of victimization by genetically related and unrelated perpetrators, respectively. Furthermore, we estimated sex differences in the etiology of emotional and sexual victimization, including larger unshared environmental influences for men and sex-limited genetic effects for women. These findings can inform child protection as they contribute to explaining why certain individuals are at increased risk of experiencing one or more types of child maltreatment.

## Introduction

The World Health Organization (2014), p. 82) defines child maltreatment as the “abuse and neglect of children under 18 years of age,” including “all types of physical and/or emotional ill-treatment, sexual abuse, neglect, negligence and commercial or other exploitation, which results in actual or potential harm to the child’s health, survival, development or dignity in the context of a relationship of responsibility, trust or power.” In line with this definition, child maltreatment is often perpetrated by parents and caregivers (Van der Kolk, 2005; Gilbert et al., 2009; National Research Council, 2014; Stoltenborgh et al., 2015). Although most modern societies have developed and implemented child welfare systems, child maltreatment remains a widespread problem exposing children worldwide to increased risk of harmful health and social consequences (Norman et al., 2012; Stoltenborgh et al., 2015; Pezzoli et al., 2019). Investigating the etiology of this phenomenon is vital as it may improve its prevention.

While previous research has examined the impact of genetic and environmental factors on the risk of perpetrating child maltreatment (e.g., Frisell et al., 2011; Långström et al., 2015; Pittner et al., 2019), the extent to which similar factors affect the risk of experiencing child maltreatment is less clear. Most often, child victimization is, in itself, conceptualized as an environmental risk factor for other detrimental outcomes, such as psychopathology (e.g., McCrory et al., 2012). In this context, genes have been shown to provide increased susceptibility to the harmful effects of child maltreatment, a phenomenon described as gene–environment interaction (GxE; e.g., Duncan and Keller, 2011; Middeldorp et al., 2014). Genes have also been found to provide a mechanism for these harmful effects, *via* changes in gene expression (i.e., epigenetic changes; e.g., Weaver et al., 2004). However, genes might also play a role in the etiology of putatively environmental variables, *via* mechanisms known as a gene–environment correlation (i.e., *r*GE, Plomin et al., 1977; Jaffee and Price, 2007; Pluess and Belsky, 2010) and genetic nurturing (Kong et al., 2018). Therefore, genetic factors might influence the risk of child victimization. Indeed, exposure to abuse or neglect within the family environment may partly depend on the genotypes that individuals within that environment possess and, to some extent, share with the victim. In line with this possibility, previous studies have estimated small to moderate genetic influences on parenting behavior and within-family conflict (Kendler and Baker, 2007), parental responsiveness, control, and physical affection (Harlaar et al., 2008), as well as child physical abuse (Sartor et al., 2012). It has also been shown that genetically influenced traits in parents and children interact bidirectionally (Larsson et al., 2008; McAdams et al., 2014), so that a child’s behavior might modulate normative parental practices, including harsh physical discipline (Jaffee et al., 2004; Avinun and Knafo, 2014). Exposure to maltreatment outside of the family environment may also partly depend on the genotype of the victim, due to genetic influences on heritable traits exploited by perpetrators. Accordingly, modest genetic influences have been estimated on adversities occurring in late childhood and not involving the family, such as difficulties in school and with peers (Johnson et al., 2013). Notably, a genetic vulnerability to experiencing child maltreatment does not mean that certain individuals are genetically predestined to be victimized. It means, instead, that certain individuals possess a genetic liability that could be compensated by protective interventions (Saudino and Hines, 2007). If the risk of victimization is increased in children with certain heritable characteristics, intervention efforts should account for this, and strategies aimed at protecting particularly vulnerable children should be developed. These interventions would be imperative for high-risk families, where both biological and social risk factors might contribute to the intergenerational cycle of maltreatment (DiLalla and Gottesman, 1991). Indeed, not all individuals who have been abused or neglected during childhood will perpetrate abuse or neglect themselves. Protective factors, such as supportive relationships with intimate partners, might prevent the reiteration of maltreatment across generations (Jaffee et al., 2013).

Despite the available evidence, it remains unclear whether gene variants and environmental exposures have a different impact on the risk of different types of maltreatment, also depending on the genetic relatedness with the perpetrator. For instance, children raised in disadvantaged family environments are more likely exposed to multiple forms of abuse and neglect (Finkelhor et al., 2007). Therefore, the etiology of multiple victimizations might be explained by genetic and environmental factors shared by siblings raised in underprivileged conditions, more than by nonshared environmental factors. As a result, preventive strategies aimed at addressing such environmental conditions might reduce the risk of multiple adversities simultaneously. On the other hand, the risk of experiencing one form of maltreatment rather than another might be influenced by environmental exposures unique to a child, more than by shared environmental factors. Furthermore, types of maltreatment like emotional and physical neglect, which are almost by definition perpetrated by parents and family members, might share etiological pathways. Instead, types of maltreatment typically involving genetically unrelated individuals, such as child sexual abuse (Sariola and Uutela, 1994; Gilbert et al., 2009), might be influenced by independent risk factors. Although investigating multiple victimizations is crucial to identify etiological influences specific and common to different types of maltreatment, the majority of previous studies have addressed childhood adversities separately (Kessler et al., 2010; Keyes et al., 2012; Armour et al., 2014). Moreover, some types of child maltreatment, such as sexual and physical abuse, have received more attention than others like emotional abuse and neglect (Norman et al., 2012; Spinhoven et al., 2014; Stoltenborgh et al., 2015). By focusing on separate types of child victimization, their relative detrimental effect, their interrelationships, and their common etiological pathways have remained largely underinvestigated. A better understanding of the co-occurrence of different types of child victimization and of the etiology of this co-occurrence is critical to guide preventive efforts.

It is also unclear whether genetic and environmental factors may have a different impact on the risk of child victimization depending on the age and sex of the child. For example, sex offenders tend to seek out pubertal more than prepubertal victims (e.g., Bergen et al., 2013; Santtila et al., 2015). As a result, genetic influences on the risk of child sexual abuse might increase from childhood to early puberty, reflecting genetic influences on the development of secondary sexual characteristics. Furthermore, heritable sex differences might explain, at least in part, sex differences in the risk of maltreatment. For example, heritable traits that are more prevalent in boys than girls, such as conduct problems, might expose boys more than girls to the risk of engaging in fights and thus of being physically assaulted (Berkout et al., 2011). Accordingly, boys are known to experience more physical abuse than girls (Thompson et al., 2004). Possible genetic sources of sex differences include the effect of genes on sex chromosomes, as well as of gene–gene interaction (epistasis) effects, when genes common to both sexes have a different effect on a trait depending on genes on the sex chromosomes. Moreover, the interplay of genes and environments might differ between sexes. In fact, girls and boys might respond to the environment differently, and at the same time, they might elicit different responses from the environment. However, sex differences have seldom been explicitly addressed in genetically informed studies (Short et al., 2013), and no previous study has investigated possible sex-limited genetic influences on the risk of child victimization. The identification of social and biological factors contributing to these sex differences is thus imperative.

Altogether, past literature suggests that early adversities might be influenced not only by environmental, but also by genetic factors. At present, however, the relative impact of these factors on the risk of child victimization is poorly understood. Therefore, in the present study, we estimated the extent of additive genetic, shared environmental, and unique environmental influences on the occurrence and co-occurrence of child emotional abuse, sexual abuse, physical abuse, emotional neglect, and physical neglect. Based on previous genetically informed studies of experienced adversities, we expected moderate genetic influences on all types of child victimization, in addition to shared and unique environmental influences. We also expected moderate genetic and shared environmental correlations between different instances of child victimization, thus explaining their covariance. Moreover, we predicted that the extent of the etiological influences would be comparable across all types of child victimization except sexual abuse. When accounting for multiple types of victimization, we further hypothesized that unique environmental exposures would predict individual types of child victimization more than genetic and shared environmental factors. After this, we tested whether the extent of genetic and environmental influences varied depending on the genetic relatedness between the victim and the perpetrator (i.e., between family-specific and family-unspecific child victimization), as well as on the sex of the victim. Due to the correlation between the genotypes of perpetrators and victims, we expected significantly larger additive genetic and smaller unique environmental influences on family-specific compared to family-unspecific child victimization. Also, based on sex differences generally observed in the prevalence of different types of child victimization, we expected significant sex differences in the magnitude of the etiological influences, possibly including sex-specific genetic effects.

## Materials and Methods

### Sample

Our sample comprised 13,024 individuals (8,415 women and 4,609 men, aged 18–49 years, mean = 29.23, *SD* = 6.82), recruited during two data collections in 2005 and 2006 through the Central Population Registry of Finland to take part in the Genetics of Sexuality and Aggression project of the Åbo Akademi University in Turku, Finland. The research plans for both major data collections were approved by the Ethics Committee of the University, in accordance with the 1964 Declaration of Helsinki. Participants provided written informed consent to their voluntary anonymous participation, *via* either a paper consent form or a secure web page. Monozygotic (MZ) and dizygotic (DZ) twins were selected for genetic analyses (n = 9,562, including 3,248 MZs and 6,314 DZs, aged 18–43 years, mean = 29.27, SD = 6.98). Zygosity was determined using standard questionnaire items addressing physical resemblance (Sarna et al., 1978) and validated through genotyping in a portion of the sample (n = 775 twin pairs). In total, 91% of this subsample was correctly classified based on questionnaire items. Demographic characteristics of our sample as well as a detailed description of the procedural aspects of the present data collection are available in Johansson et al. (2013).

### Measures and Preliminary Data Handling

We collected retrospective accounts of child victimization using the Childhood Trauma Questionnaire Short Form (CTQ-SF; Bernstein et al., 2003). The CTQ-SF measures five types of maltreatment by family members or others when the respondent was “growing up” and thus irrespective of the age of victimization. The CTQ-SF has demonstrated reliability and validity in clinical and community-based samples (Scher et al., 2001). Participants rated the frequency of 25 instances of emotional abuse, physical abuse, sexual abuse, emotional neglect, and physical neglect on a five-point scale, ranging from “never true” to “very often true.” Three additional items, aimed at detecting socially desirable attitudes, were not used. Responses to seven items were reverse-coded, and 1.5% (SD = 1.9, min = 1.2, max = 2.2) of responses were imputed using the expectation maximization procedure. Items were also transformed with log10 transformation to correct for nonnormality. After transformation, most values were within the acceptable range (skewness <|3|; kurtosis <|10|; Kline, 2015). Items were regressed on age and sex, to control for these common sources of systematic variance, except when addressing sex differences. In this case, items were regressed on age only.

### Statistical Analyses

Statistical analyses included phenotypic and quantitative genetic analyses. At the phenotypic level, we examined the mean scores on all items in the full sample as well as in subsamples by sex, and we estimated their zero-order correlation coefficients. Then, we created five-factor scores, corresponding to the five types of child victimization examined and consistent with the validated five-factor structure of the CTQ-SF (Spinhoven et al., 2014). To do so, we employed dimension reduction by maximum likelihood factor analysis, extracting a single factor and saving factor scores using the Bartlett method. We also categorized items as “family-specific” or “family-unspecific” based on face validity, i.e., based on whether they explicitly referred to parents, family members, and family conditions (e.g., “I felt that someone in my family hated me,” and “I did not have enough to eat”) or, instead, to undefined individuals (e.g., “Someone molested me”). After this, we created two mean scores by averaging responses to items addressing family-specific and family-unspecific victimization, respectively, and standardized them as *z* scores. Next, we examined the cross-twin cross-trait, cross-twin within-trait, and within-twin cross-trait correlations between all scores in MZ and DZ twins separately. At the quantitative genetic level, we tested univariate, bivariate, and multivariate twin models. Using univariate models, we decomposed the variance of all scores of child victimization into additive genetic (A), shared environmental (C), and unique environmental (E) influences, in the full sample as well as in subsamples by sex. Then, we compared the magnitude of the etiological influences across the five types of child victimization, between family-specific and unspecific victimization, and between women and men. Using bivariate models, we estimated the genetic (*r*G), shared environmental (*r*C), and unique environmental (*r*E) contribution to the correlation between the five types of child victimization. Using a multivariate common pathway model, we further estimated one latent factor accounting for the covariance of the five types of child victimization, to inspect possible changes in the etiological pathways when accounting for multiple victimizations. Lastly, using sex limitation models, we investigated whether the same sets of genes influenced the risk of child victimization in women and men. Of note, across twin models, unique environmental terms also included variation due to measurement error. More information on how to estimate these twin models can be found in Neale and Cardon (2013).

Absolute and relative model fit indices were used for model acceptance. Among the indices of global fit, we used the root mean square error of approximation (RMSEA), with upper 90% confidence interval (CI) values smaller than 0.08, indicating mediocre fit and smaller than 0.06 indicating excellent fit (Hu and Bentler, 1999). Among the indices of relative fit, we used the Akaike Information Criterion (AIC; Akaike, 1987) and the Bayesian Information Criterion (BIC; Raftery, 1995), with lower values indicating a better tradeoff between model fit and model complexity. To compare nested sex limitation models, we used the Satorra-Bentler scaled χ^2^ difference test (Satorra and Bentler, 2010). Standardized measures of effect size (ES) (Cohen *d*; Cohen, 1988) were computed for significant comparisons between groups of observations. Moreover, we examined whether any differences in the magnitude of the A, C, and E estimates, observed between types of child victimization or groups of participants, were significant. To do so, we calculated the 95% CIs around the estimates based on bootstrapped standard errors (1,000 resamples with replacement). Then, we inspected whether the CIs overlapped. No overlap or CIs just touching were considered indicative of a significant difference (*p* < 0.01, Cumming and Finch, 2005). Unstandardized measures of ES, based on raw differences between individual estimates, are presented for significant comparisons (Kelley and Preacher, 2012).

### Software

SPSS Statistics for Macintosh, 23.0 (IBM, 2015) was used for preliminary data handling. Analyses were performed in Mplus 8 (Muthe´n and Muthe´n, 1998–2017) and R environment for statistical computing 3.3.2 (R Core Team, 2016), package OpenMx 2.10.0 (Neale et al., 2016).

## Results

### Phenotypic Results

**Figure 1:**
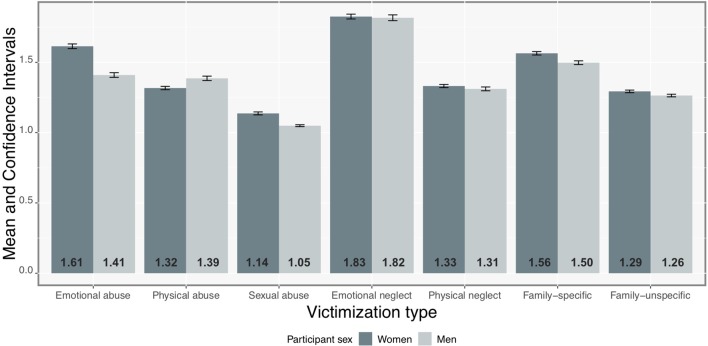
Bar graph reporting average scores of child victimization in two subsamples by sex.

Mean levels for the different types of victimization are depicted in **Figure 1**. Compared to the remaining types of child victimization, participants reported significantly higher mean levels of emotional neglect (*p* < 0.01, *d* = 0.64) and emotional abuse (*p* < 0.01, *d* = 0.12), as well as significantly lower levels of sexual abuse (*p* < 0.01, *d* = 0.33). Moreover, compared to women, men reported significantly lower mean levels of emotional abuse (*p* < 0.01, *d* = 0.18) and sexual abuse (*p* < 0.01, *d* = 0.04), as well as significantly higher mean levels of physical abuse (*p* < 0.01, *d* = 0.05). Participants reported significantly higher mean levels of family-specific than family-unspecific victimization (*p* < 0.01, *d* = 0.07).

Item-level zero-order correlation coefficients were positive. On average, correlations between items addressing the same type of child victimization were moderate (*r*_mean_ = 0.45, SD = 0.02), whereas correlations between items addressing different types of child victimization were small (*r*_mean_ = 0.22, SD = 0.02). In other words, participants with a history of child victimization showed a moderate tendency to report multiple instances of the same type of abuse or neglect and, in addition, a small tendency to report other types of abuse and neglect. Items addressing child sexual abuse were the most strongly correlated with each other (*r*_mean_ = 0.58, SD = 0.09) and the least strongly correlated with the remaining items (*r*_mean_ = 0.15, SD = 0.05). Thus, participants with a history of child sexual victimization likely experienced multiple instances of sexual abuse, but rarely in the context of other forms of abuse and neglect. Altogether, the pattern of item-level correlations supported the presence of five types of child victimization. By inspecting the determinants of the correlation matrices, we further excluded extreme collinearity and singularity (Field, 2013), and then, we performed dimension reduction. The five resulting factor scores showed good or acceptable internal consistency (Cronbach α: emotional abuse = 0.81, physical abuse = 0.76, sexual abuse = 0.87, emotional neglect = 0.86, physical neglect = 0.65). Factor loadings were moderate to strong on average (mean = 0.67, SD = 0.11), strongest for items inquiring sexual abuse (mean = 0.76, SD = 0.09) and weakest for those inquiring physical neglect (mean = 0.52, SD = 0.07).

Phenotypic twin correlations were also calculated between all composite scores. On average, cross-twin within-trait correlations were larger in MZs than in DZs (*r*_mean_ = 0.58 vs. *r*_mean_ = 0.35). Given the greater genetic similarity between MZs compared to DZs, this result was indicative of additive genetic influences, irrespective of the type of child victimization and the genetic relatedness with the perpetrator. However, DZ correlations slightly larger than half the MZ correlations implied shared environmental influences. Also, MZ correlations smaller than unity were suggestive of unique environmental influences as MZs share, by definition, genetic and shared environmental background. Lastly, average cross-twin cross-trait correlations were larger in MZs than in DZs (*r*_mean_ = 0.31 vs. *r*_mean_ = 0.19), indicating genetic influences common to different types of child victimization.

### Genetic Results

#### Univariate Models

Univariate twin models were estimated in order to inspect whether the extent of the etiological influences varied as a function of the type of child victimization experienced, the genetic relatedness between the victim and the perpetrator (i.e., a parent or family member, vs. undefined individuals), as well as the sex of the victim (see **Table 1** for standardized estimates and model fit indices from the best-fitting models). Additive genetic influences were moderate on emotional abuse, sexual abuse, and emotional neglect and were modest on physical abuse and neglect. Shared environmental influences were modest on physical neglect, small on physical abuse and emotional neglect, and nonsignificant for emotional and sexual abuse. Unique environmental influences were moderate on all types of child victimization. Shared and unique environmental influences differed significantly between family-specific and family-unspecific items. Specifically, shared environmental influences reached significance only for family-specific victimization, whereas unique environmental influences were significantly larger for family-unspecific victimization (*p* < 0.01, ES = 0.11). Unique environmental influences were significantly larger in men compared to women for emotional abuse (*p* < 0.01, ES = 0.10), sexual abuse (*p* < 0.01, ES = 0.29), and emotional neglect (*p* < 0.01, ES = 0.24). No other significant sex difference emerged.

**Table 1:** Estimates and model fit statistics, best-fitting univariate twin models.

	Cross-twin correlations		Standardized Squared Estimates [95% CIs] *									Model fit Indices			
			Full sample			Women			Men						
	MZ	DZ	A	C	E	A	C	E	A	C	E	Chi2 (df) *	RMSEA [95%CIs]	AIC	BIC
EA	0.59	0.33	.61 [.58, .64]		.39 [.35, .43]	.65 [.62, .68]		.35 [.31, .39]	.52 [.44, .60]		.48 [.40, .57]	11.78 (7)	.01 [.00, .03]	28272.47	28292.88
PA	0.56	0.39	.33 [.09, .56]	.22 [.04, .41]	.45 [.38, .51]	.30 [.18, .42]	.28 [.02, .54]	.41 [.34, .49]	.32 [.01, .63]	.25 [.21, .29]	.43 [.33, .53]	5.37 (6)	.00 [.00, .02]	28555.29	28582.51
SA	0.47	0.20	.49 [.39, .59]		.51 [.41, .61]	.59 [.51, .68]		.41 [.30, .52]	.24 [.16, .32]		.76 [.60, .92]	101.43 (7)	.06 [.05, .07]	27539.68	27560.09
EN	0.60	0.40	.42 [.33, .50]	.19 [.08, .30]	.40 [.36, .43]	.37 [.23, .50]	.29 [.16, .42]	.35 [.30, .39]		.38 [.32, .43]	.62 [.58, .66]	23.01	.03 [.02, .04]	27547.53	27574.75
PN	0.61	0.44	.26 [.13, .40]	.32 [.23, .42]	.41 [.37, .45]	.14 [.02, .26]	.47 [.36, .57]	.39 [.34, .43]	.53 [.45, .61]		.47 [.39, .55]	7.21 (6)	.02 [.00, .03]	30168.96	30196.17
FSP	0.68	0.43	.47 [.39, .56]	.20 [.09, .31]	.33 [.29, .36]	.44 [.31, .57]	.34 [.21, .48]	.32 [.27, .36]	.33 [.05, .60]	.25 [.23, .27]	.43 [.36, .50]	10.64 (6)	.02 [.00, .03]	25979.98	26007.2
FUNSP	0.54	0.30	.57 [.51, .62]		.44 [.37, .50]		.27 [.12, .42]	.73 [.64, .82]	.48 [.38, .57]		.52 [.43, .61]	22.83 (7)	.03 [.02, .04]	26269.19	26289.56

Cross-twin correlations and model fit indices were estimated in the full sample. Nonsignificant estimates were constrained to zero and are thus omitted. *p < 0.01. EA, emotional abuse; SA, sexual abuse; PA, physical abuse; EN, emotional neglect; PN, physical neglect; FSP, family-specific victimization; FUNSP, family-unspecific victimization; MZ, monozygotic twin pair; DZ, dizygotic twin pair; A, additive genetic influences; C, shared environmental influences; unique environmental influences; RMSEA, Root Mean Square Error of Approximation; AIC, Akaike Information Criterion; BIC, Bayesian Information Criterion.

#### Genetic Correlations

Using bivariate twin models, we analyzed the genetic and environmental decomposition of the correlations between the five types of child victimization, to clarify whether similar influences contributed to their co-occurrence. On average, genetic and shared environmental correlations were positive and moderate to large (*r*G_mean_ = 0.43, SD = 0.27; *r*C_mean_ = 0.55, SD = 0.65), whereas unique environmental correlations were positive but small (*r*E_mean_ = 0.13, SD = 0.10). In other words, genetic and shared environmental risk factors contributed to the co-occurrence of individual instances of child victimization more than unique environmental risk factors. Bivariate correlations were substantially consistent across sexes.

#### Multivariate Common Pathway Model

**Figure 2:**
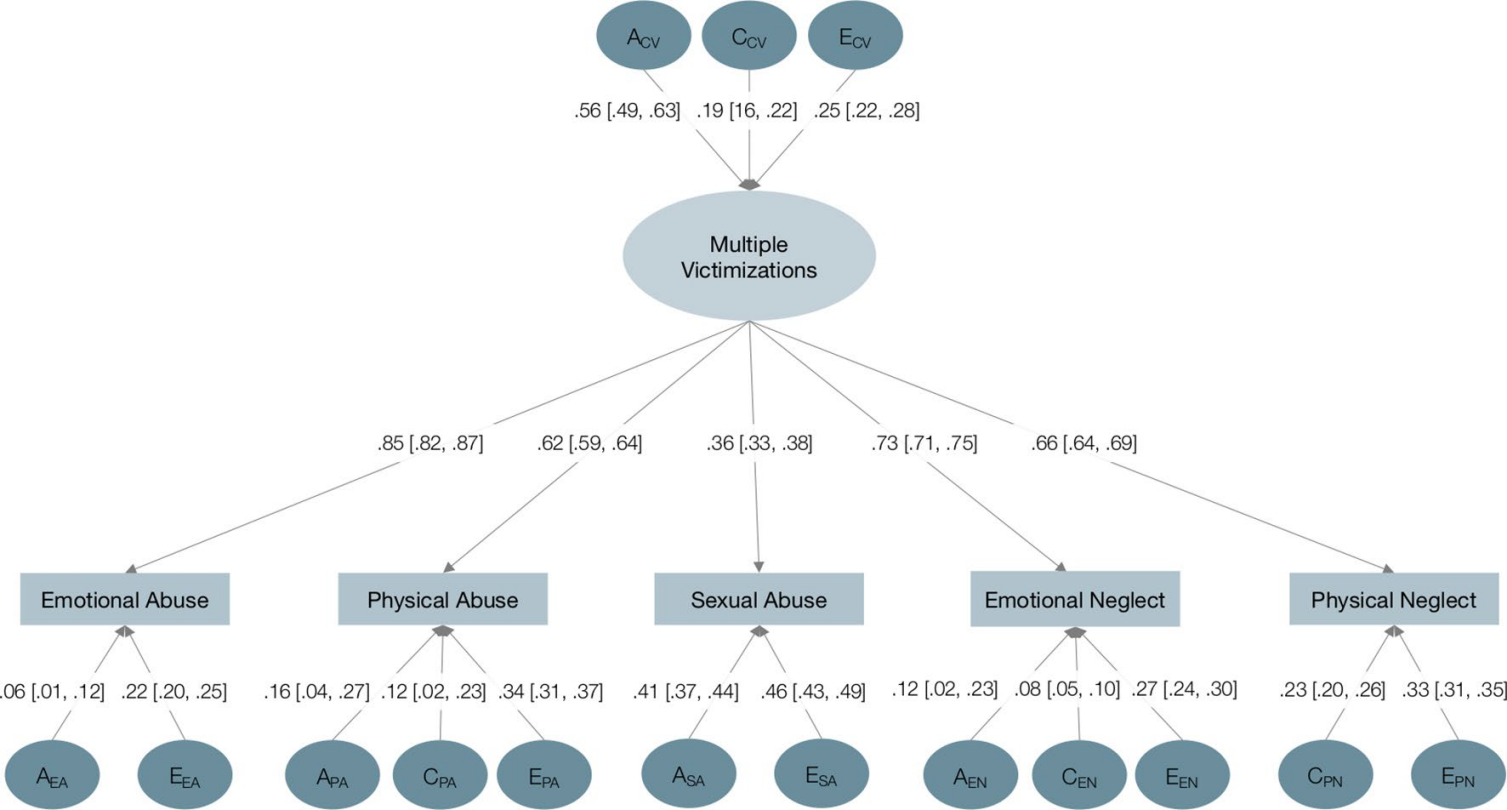
Multivariate common pathway twin model of multiple child victimizations. *A*, additive genetic influences; *C*, shared environmental influences; *E*, unique environmental influences. Squared standardized estimates (i.e., variance components) and standardized factor loadings are reported on the corresponding paths, confidence intervals are reported in brackets. Nonsignificant paths were constrained to zero and are thus omitted.

We further estimated one latent factor, or common pathway, accounting for the covariance between the five types of child victimization, and we decomposed its variance into A, C, and E components (**Figure 2**). This model fitted our data excellently (χ^2^ = 1116.34, *df* = 100, *p* < 0.01, RMSEA = 0.06, 90% CIs = 0.05, 0.06, AIC = 129,767.13, BIC = 129,971.238). In the common pathway model, compared to the univariate models, we estimated smaller additive genetic influences on all types of abuse, significantly for emotional abuse (*p* < 0.01, ES = 0.50) and emotional neglect (*p* < 0.01, ES = 0.29), but inappreciably for sexual abuse. Furthermore, we estimated significantly increased unique environmental influences on all types of child victimization (*p* < 0.01; ES = 0.17 for emotional abuse; ES = 0.11 for physical abuse; ES = 0.13 for emotional neglect; ES = 0.10 for physical neglect), except for sexual abuse. No significant difference emerged between models in the shared environmental influences, which were substantial only for physical neglect and small or negligible for the remaining types of child victimization. Altogether, multivariate analyses supported the presence of common genetic influences underlying the risk of emotional victimization and other types of child victimization, as well as distinct genetic and unique environmental influences underlying the risk of sexual abuse. While genetic factors largely influenced the susceptibility to multiple victimizations, unique environmental factors largely influenced the likelihood of experiencing one type of child victimization rather than others. Moreover, shared environmental influences were small overall and unchanged when accounting for multiple types of victimization.

#### Sex Limitation Models

Lastly, to clarify whether not only the magnitude of the etiological influences differed between sexes, but also whether different sets of genes influenced the risk of child victimization in women and men, we tested a series of sex limitation models. Two models were estimated for each type of child victimization: a baseline, or “general” model, and a restricted, or “common effects” model. In the general model, the A, C, and E variance components of each composite score were allowed to differ between sexes, and one additional component was estimated (*A*’), corresponding to the potential sex-specific set of genes. In the common effects model, the sex-specific component was fixed to zero, thus estimating only genetic effects common to both sexes. For each type of child victimization, the common effects model was then tested against the general model, to determine statistical significance of the sex-specific component. The difference in fit was close to significance only for emotional abuse, χ^2^(2, n = 9,152) = 5.96, *p* = 0.051, and sexual abuse, χ^2^(2, n = 9,152) = 5.85, *p* = 0.054. In particular, for emotional abuse, sex-limited genetic effects exerted a significantly larger impact on women than men (*A*’^2^ = 0.45, SE = 0.07, vs. *A*’^2^ = 0.15, SE = 0.05; *p* < 0.01, ES = 0.31), whereas the opposite was true for genetic influences common to both sexes (*A* ^2^ = 0.29, SE = 0.04, vs. *A* ^2^ = 0.54, SE = 0.05; *p* < 0.01, ES = 0.24). For sexual abuse, instead, no significant difference emerged in the magnitude of the genetic influences common to both sexes, but the sex-limited genetic path was nonsignificant for men (*A*’^2^ = 0.01, SE = 0.23, *p* = 0.66), suggesting the presence of specific sets of genes influencing the risk of sexual abuse in women only. If replicated, these results would indicate that different sets of genes might influence emotional and sexual abuse in girls and boys.

## Discussion

### Etiological Pathways Underlying Single and Multiple Types of Child Victimization

In the present study, we investigated the etiology of the occurrence and co-occurrence of emotional abuse, physical abuse, sexual abuse, emotional neglect, and physical neglect, in a large population-based sample. In line with previous studies (see Stoltenborgh et al., 2015, for a meta-analysis), we estimated higher mean levels of emotional abuse and neglect, as well as lower mean levels of sexual abuse, compared to other types of child victimization. While assuming that all types of child victimization deserve proportionate empirical attention and preventive efforts, this finding motivates further research on the characteristics and consequences of child emotional abuse and neglect, which have been somewhat overlooked by previous research (e.g., English et al., 2015). After this, we estimated the magnitude of the genetic and environmental influences specific and common to multiple types of child victimization. Our results indicated that the risk of child victimization might be influenced by genetic factors, in addition to environmental factors. Moreover, the risk of multiple victimizations might be influenced by genetic and shared environmental more than nonshared environmental factors. Instead, the risk of experiencing one form of victimization rather than another might be largely influenced by unique environmental factors, including intrasubject variation but also measurement error, possibly elevated given the retrospective nature of our self-reports. When accounting for multiple types of victimization, we were able to isolate distinct genetic and unique environmental risk factors for sexual abuse. In addition, we examined whether the impact of genetic and environmental influences varied as a function of the genetic relatedness between the victim and the perpetrator. Our results suggested that shared and unique environmental factors might influence the risk of victimization by related and unrelated individuals, respectively.

These findings have implications for both empirical and child protection practices. As child victimization is, at least in part, influenced by genes, researchers should attempt to account for genetic confounding, when estimating its phenotypic association with potential outcomes (e.g., Barbaro et al., 2017). From a practical standpoint, the fact that children who experience one type of victimization are at heightened risk of experiencing others, especially due to genetic and shared environmental mechanisms, should guide initiatives to protect vulnerable children. In fact, our findings indicate that successful prevention of child abuse and neglect necessarily entails identifying and supporting families where parental risk factors for child maltreatment are present. Moreover, unique situational factors that might increase the risk of sexual abuse should be identified. During childhood, unique environmental exposures frequently correspond to exposures outside the family environment, where this type of abuse is most frequently perpetrated (Sariola and Uutela, 1994; Tadei et al., 2017). Nonetheless, familial risk factors should also be examined. Furthermore, given the evidence of genetic influences on sexual abuse, future research should clarify whether and which heritable traits might increase the risk of being taken advantage of sexually motivated perpetrators. In fact, a precise characterization of personal and contextual risk factors for child sexual abuse is crucial to implement adequate preventive strategies. Lastly, as the risk of victimization by genetically unrelated individuals might be especially influenced by unique environmental exposures, further research should investigate possible clusters of extrafamilial risk factors, to better understand which situations increase the risk of victimization outside the household.

### Sex Differences in the Etiology of Child Victimization

Sex differences emerged both in the mean levels of the reported adversities as well as in the etiological influences on them. Despite such differences, the phenotypic patterns of co-occurrence were similar and influenced by similar factors. In line with previous studies (Thompson et al., 2004; Stoltenborgh et al., 2011), we observed higher mean levels of emotional and sexual abuse in women and higher mean levels of physical abuse in men. Consistent with previous evidence of the comparable distribution of other forms of child victimization between sexes (Jud et al., 2016), no other significant difference emerged. Moreover, we expanded on previous research by investigating the extent of the etiological factors contributing to sex differences in the risk of child victimization. First, we estimated smaller unique environmental influences in women, compared to men, for emotional abuse, sexual abuse, and emotional neglect. Although we can only speculate on the reasons for this result, one possibility is that girls are exposed to less variation in the unique environmental risk factors for these forms of child victimization, because gender-based psychological violence and sexual violence are more commonplace among them (Ellsberg et al., 2015). As a result, the relative impact of unique environmental factors might appear reduced. Boys, on the other hand, might be exposed to more variation in unique environmental risk factors. For example, their unsafe environmental exposures might vary, depending on individual differences in sensation seeking (Cross et al., 2013), or in the tendency to conform with social expectations of audaciousness (Doey et al., 2014). As a result, the relative impact of unique environmental factors might appear increased. Second, we found preliminary evidence that different sets of genes might influence the risk of emotional and sexual abuse in girls and boys. Specifically, sex-limited genetic effects might have a significantly greater impact on the risk of emotional and sexual abuse in girls. Thus, in our sample, sex-limited genetic influences might have contributed to the significant sex differences observed in the rates of emotional and sexual abuse. More broadly, sex differences in the risk factors for child victimization, including differences in heritable traits as well as in the environments that boys and girls tend to be exposed to, suggest the importance of sex-specific prevention strategies.

### Limitations

Our results should be interpreted in the context of five main limitations. First, the study employed only one psychometric scale. Although we addressed more than just one type of child victimization, not all possible covarying exposures were included. It is therefore unclear whether the same findings would be observed using different measurement tools. Second, we employed a retrospective self-report measure. Although empirical evidence supports the consistency of retrospective and prospective self-reports of child victimization (Hardt and Rutter, 2004; Reuben et al., 2016), retrospective reports may inflate unique environmental estimates, which include measurement error. Furthermore, due to heritable differences in memory ability, retrospective reports may inflate additive genetic estimates. Third, our items did not measure the exact age at which child victimization had occurred. Therefore, we could not address whether the etiology of child victimization varied also depending on the age of the child. This limitation is especially critical, as the type, severity, and impact of abuse and neglect might differ over developmental stages. For example, since intrafamilial compared to extrafamilial child sexual abuse has an earlier age at onset (Ventus et al., 2017), shared environmental factors might influence the risk of this type of abuse in early more than in late childhood. In addition, since autobiographical memory functioning increases over developmental stages (Willoughby et al., 2012), earlier experiences might be remember less accurately than more recent ones, thus increasing the likelihood of recollection bias. Fourth, how well the findings generalize to nontwins and families without twins is unclear. The magnitude of the simultaneous parental investment is larger for parents of twins compared to parents of age-discrepant siblings. This could increase, for example, parental stress (Thorpe et al., 1991), which might constitute risk factors for child victimization (Yokoyama et al., 2015). Lastly, although the current sample is highly representative of the Finnish population (Johansson et al., 2013; Rehan et al., 2017), some cultural aspects pertinent to child victimization might not generalize to other populations. In particular, studies using similar operational definitions suggest lower rates of child maltreatment in Finland (Fagerlund et al., 2014), compared to other European countries (Bellis et al., 2013), but a similar declining trend during the last decades (e.g., Laaksonen et al., 2011; Jud et al., 2016).

### Conclusion

The current study demonstrated that the risk of child victimization might be influenced by genetic factors, presumably *via r*GE mechanisms. Heritable characteristics in the child, as well as features of the rearing environment, were found to especially influence the co-occurrence of multiple types of child victimization. Moreover, genetic and environmental factors were found to play a different role in the etiology of intrafamilial and extrafamilial child victimization. As a result, both individual and familial risk factors should be addressed to develop adequate preventive strategies. Furthermore, after accounting for multiple types of victimization, unique environmental factors still influenced the risk of specific types of child victimization. In particular, specific etiological pathways emerged for sexual abuse, indicating the need for more genetically informed investigations of this phenomenon and its etiology as distinct from other forms of victimization. Lastly, we estimated substantial sex differences in the etiology of child victimization, including sex-limited effects on the risk of emotional and sexual abuse, which might contribute to sex differences in the prevalence rates of child victimization and in the susceptibility to its harmful consequences.

## Data Availability

The scripts coded for statistical analyses with their respective outputs are available at the project site on Open Science Framework (OSF, osf.io/akrjc). The dataset is available upon request, in line with the decision of our ethics committee.

## Ethics Statement

This study was carried out in accordance with the recommendations of the Board for Research Ethics at Åbo Akademi University. All subjects gave written informed consent in accordance with the Declaration of Helsinki. The protocol was approved by the Board for Research Ethics at Åbo Akademi University.

## Author Contributions

PS, PP, JA, and AH contributed to the conceptualization of the study; PP and AH contributed to methodology and formal analyses; PP contributed to writing and editing the original draft; JA and PS contributed to supervision and review; PS, JA, and PP contributed to funding acquisition.

## Funding

This work was supported by the Academy of Finland (http://www.aka.fi/en), grants 287800 (to PS) and 298513 (to JA), as well as by a personal grant of the Research Foundation of the Mannerheim League for Child Welfare (https://www.mll.fi; PP). The funders had no role in study design, data collection and analysis, decision to publish, or preparation of the manuscript.

## Conflict of Interest Statement

The authors declare that the research was conducted in the absence of any commercial or financial relationships that could be construed as a potential conflict of interest.
